# Support through Social Media and Online Class Participation to Enhance Psychological Resilience

**DOI:** 10.3390/ijerph182211962

**Published:** 2021-11-14

**Authors:** Muhammad Zaheer Asghar, Seema Arif, Elena Barbera, Pirita Seitamaa-Hakkarainen, Ercan Kocayoruk

**Affiliations:** 1 Department of Education, University of Helsinki, 00014 Helsinki, Finland; pirita.seitamaa-hakkarainen@helsinki.fi; 2 School of Doctorate, Education & ICT (e-Learning), Universitat Oberta de Catalunya, 08018 Barcelona, Spain; 3 Department of Education, University of Management and Technology, Lahore 54770, Pakistan; drseema.arif@gmail.com; 4 Faculty of Psychology and Education Sciences, Universitat Oberta de Catalunya, 08018 Barcelona, Spain; ebarbera@uoc.edu; 5 Faculty of Education, Çanakkale Onsekiz Mart University, Çanakkale 17100, Turkey; kocayoruk@comu.edu.tr

**Keywords:** COVID-19 lockdown, social media, social support, online class participation

## Abstract

Social support was an important factor in minimizing the effect of social isolation during the COVID-19 pandemic lockdown. This research aimed to study the role of online class participation and social media usage to link the social support available from family and friends to psychological resilience among pre-service special education teachers against the negative psychological effects of the COVID-19 pandemic lockdown. A survey was conducted with 377 pre-service special needs education (SNE) teachers enrolled at universities in Pakistan. Partial least square structural equation modeling (PLS-SEM) was applied using Smart-PLS 3.2.8. Results revealed that social media and online class participation played a mediating role between social support and psychological resilience in the pre-service special needs education (SNE) teachers studied. Teacher education institutions can devise strategies to develop social media platforms for student socialization during an emergency to help build resilience against the negative psychological effects of social isolation. Future studies could be conducted to adapt instructions and curricula to social media environments for education in an emergency.

## 1. Introduction

Universities across the globe shifted from face-to-face learning to online learning during the COVID-19 lockdown [1,2,3]. This shift increased social isolation, resulting in several psychological problems among higher education students, such as anxiety, stress, and depression [4]. Students themselves or their families and friends have been adversely affected by the spread of the pandemic. They have witnessed death and disease all around them; many believed that their survival and well-being were in jeopardy. Pakistan suffered through lesser loss compared with the rest of the world. However, that does not mean that our normal life and social interaction were not disturbed during the strict lockdown imposed during March–October 2020. Pakistanis are gregarious people who love spending time in the company of family and friends. Not surprisingly then, social support from friends and family was an important factor in the psychological well-being of the students. 

People perceive the care and support provided by others as social support [5]. The COVID-19 situation was stressful because it not only affected the vulnerable but was a new kind of disease spread by a virus that was treated only symptomatically. The COVID-19 pandemic and lockdown led to social isolation and negative feelings such as stress, anxiety, and depression among students [6]. Research has shown that social support reduces stress, minimizes the effect of stressful situations, prepares individuals in difficult conditions, and enhances their coping abilities [7]. The significance of social support for its positive influence on people’s happiness and health was accepted long ago, and perceived social support has an important impact on individuals by enhancing their psychological and physical health [7,8]. Social support most importantly functions as a buffer to reduce or balance the psychological effects of a stressful event. Several studies have found that social support boosts psychological resilience, helping to decrease stress and increase psychological health [9,10]. 

Resilience during the COVID-19 pandemic crisis is defined as appropriate adaptation to stressors and the process of rapid recovery, especially when individuals need social support in the period of crisis [11]. Researchers [12] presented a model of resilience that represents individual resilience in a community as a product of networked resources such as social capital, economic development, and communication of information. According to researchers [13,14], more resilient communities can be developed by creating new horizons of collaboration using social media tools in response to crises. Studies [15] state that the significance of online platforms in connecting individuals with communities and strengthening connectivity has been observed in communities during emergencies [14], and this helps to develop effective and strong offline interactions and networks [16,17]. Researchers [18] and [11] point out that online networks, collaboration, and social support are also important to relieve the stress that follows crises.

There were two major sources of online socialization available for higher education students in Pakistan during the COVID-19 lockdown and social isolation. The first was online class participation, which helped students to interact with their classmates, teachers, and mentors. The second was social media networks, which connected the students with their friends and other members of society. Online platforms, either in formal online class participation or informal interaction on social media, connected individuals with an online community that helped them overcome distance and time barriers to connect and reconnect with other people [16,17,19]. For all these reasons, online platforms seem to have the ability to significantly increase resilience among students during crises. Online socialization platforms in the form of social media, as well as online class participation, protect students against the possible threats of depression, stress, and anxiety usually experienced by individuals during a state of emergency, and they increase people’s well-being [19]. Research has also provided empirical evidence of the influence of online interaction as a valuable alternative to face-to-face communication and of the accompanying social, economic, and health benefits [16]. Nevertheless, in an emergency such as the COVID-19 pandemic lockdown, the shift from face-to-face classes to online classes and social media’s role in providing social support from friends and family to bolster students’ psychological resilience is an understudied phenomenon. There are studies available on different aspects of resilience among children and other strata of society, but the research remains scant regarding psychological resilience among higher education students [20]. 

Lockdown and social isolation increased threats to individuals’ psychological health, and while the psychological well-being of health workers is considered essential [21], people working in the field of special education cannot be ignored. Teachers and other special education staff already have difficulty communicating with their students, and the COVID-19 pandemic proved to be disastrous in this sense. The workload of health workers and special needs education (SNE) teachers increased due to the spread of COVID-19 [22], and there was a shortage of available resources. Fear of contracting the coronavirus, social isolation, and work pressure took a toll on the mental health and psychological well-being of individuals in the special needs education sector. In this unique study, we focused on special education students and pre-service SNE teachers with a background as health workers. The aim was to understand the mediating role of online platforms (i.e., social media and online class participation) in the relation between social support (i.e., support from family and friends), and psychological resilience in pre-service special education teachers in Pakistan. 

The following three research questions were posed:

RQ1. What was the impact of social support (i.e., support from family and friends) on online class participation and social media use among pre-service SNE teachers during the COVID-19 lockdown?
RQ2. What effect did online platforms (i.e., social media and online class participation) have on psychological resilience among pre-service SNE teachers during the COVID-19 lockdown?
RQ3. What was the mediating role of online platforms (i.e., social media and online class participation) in the relation between social support (i.e., from family and friends) and psychological resilience in pre-service SNE teachers during the COVID-19 lockdown?

This paper begins with a literature review, followed by hypotheses development, and a description of the research methodology. Data analysis was performed to obtain the results, which are discussed in the light of the current literature. The conclusions and implications of the study are provided at the end of this paper. 

## 2. Literature Review

Psychological resilience is defined as the ability to respond to and overcome extremely stressful or traumatizing and adverse life experiences [4,23]. The relationship between emergency situations such as a pandemic and the resultant psychological distress is well established [5]. High levels of perceived psychological distress during the COVID-19 pandemic have been proven by recent research, and the coronavirus pandemic has posed huge challenges for the psychological resilience of people worldwide [24]. Researchers [25] identified psychological resilience and coping behavior as essential strategies to facilitate an individual’s ability to rebound positively and cope with complex situations, traumatic events, and hardships in order to boost psychological well-being.

Researchers [26] reported that there is a correlation between social support and psychological well-being during disasters and emergencies. The authors of [27] highlighted that previous studies have identified psychological resilience, coping strategies, and social support as a protective shield against stress, anxiety, and burnout among nursing staff and allied teachers caring for people with special needs during disease outbreaks such as MERS-CoV, Ebola, and SARS. Another study [28] showed the same patterns of coping behavior, psychological resilience, and social support among allied staff working with people with disabilities on the frontline during the COVID-19 pandemic.

The authors of [26] mentioned that emergency situations such as disasters, disease outbreaks, and calamities trigger adverse psychological problems when those affected are not provided with social and psychological support. Support from family, friends, colleagues, and peers plays an important role in sustaining a healthy balance of emotions. Social support is described as the availability of trusted people who remind us that they care about, respect, and value us. Social support includes both the perceived support and received support that helps to increase psychological well-being [5,29]. Perceived support is a person’s subjective appreciation of the accessible and appropriate resources and responses provided by their social circle. Received social support is the objective appreciation of a person’s social links and their functioning. There are different sources of social support, including friends, family, colleagues, and peers. Social links provide an awareness of different products and resources that are helpful to change behavior. Research has revealed the importance of perceived social support as positive psychology to enhance self-esteem and resilience [30,31]. For the purposes of this study, we have divided social support into two categories. The first concerns the family support, which was available to students directly while they were confined in their homes. The second category includes support provided by friends through online platforms such as social media networks and online class participation.

The hypotheses arising from the literature are as follows:

**Hypothesis** **1** **(H1a).** *Social support from friends had a significant positive effect on the psychological resilience of pre-service special education teachers during the COVID-19 pandemic.*

**Hypothesis** **1** **(H1b).** *Family social support had a significant positive effect on the psychological resilience of pre-service special education teachers during the COVID-19 pandemic.*

The biggest challenge facing higher education during the COVID-19 lockdown and shift to online classes was preventing students from dropping out. According to a study [32], friends and family support is an important factor for preventing students from dropping out of online classes. Families who are very enthusiastic about their children’s education encourage them to participate in online classes in a serious effort to improve their learning [32]. Meanwhile, the presence of family and friends on social media platforms also encourages people to use social media during an emergency to connect with society. The hypotheses that arise are as follows:

**Hypothesis** **2** **(H2a).** *Families provided social support for students to encourage online class participation during the COVID-19 lockdown.*

**Hypothesis** **2** **(H2b).** *Friends provided social support for students to encourage online class participation during the COVID-19 lockdown*.

**Hypothesis** **2** **(H2c).** *Social media was a source of family support for students during the COVID-19 lockdown.*

**Hypothesis** **2** **(H2d).** *Social media was a source of friends support for students during the COVID-19 lockdown.*

A study [33] reported a growing trend of conducting educational programs using e-learning tools such as LMS, university Skype, Blackboard, and social media such as WhatsApp, which is used for informal interaction between students and teachers. Since the advent of 4G internet services, the number of social media users has grown exponentially. Government policies worldwide have been promoting the use of multimedia applications for educational purposes [34], especially during the pandemic, creating innovative education models and endorsing problem-based learning on social network sites for student learning and socialization [35]. Online platforms not only help to continue education in emergencies, they also helped to boost students’ psychological resilience against the negative psychological effects of the COVID-19 lockdown. 

Researchers have also investigated whether online class participation, social media usage, or both have the potential to reduce levels of depression and boost the perceived social support and self-esteem of undergraduate students [33]. Results show that the use of online platforms such as professional learning sites and social media networks significantly decreases depression and loneliness and increases self-esteem and perceived social support [36,37]. The hypotheses raised from the discussion are as follows:

**Hypothesis** **3** **(H3a).** *Online class participation played a mediating role between social support from family and psychological resilience during the COVID-19 lockdown.*

**Hypothesis** **3** **(H3b).** *Online class participation played a mediating role between social support from friends and psychological resilience during the COVID-19 lockdown.*

**Hypothesis** **4** **(H4a).** *Social media played a mediating role between social support from friends and psychological resilience during the COVID-19 lockdown.*

**Hypothesis** **4** **(H4b).** *Social media played a mediating role between social support from family and psychological resilience during the COVID-19 lockdown.*

### Conceptual Framework

**Figure 1:**
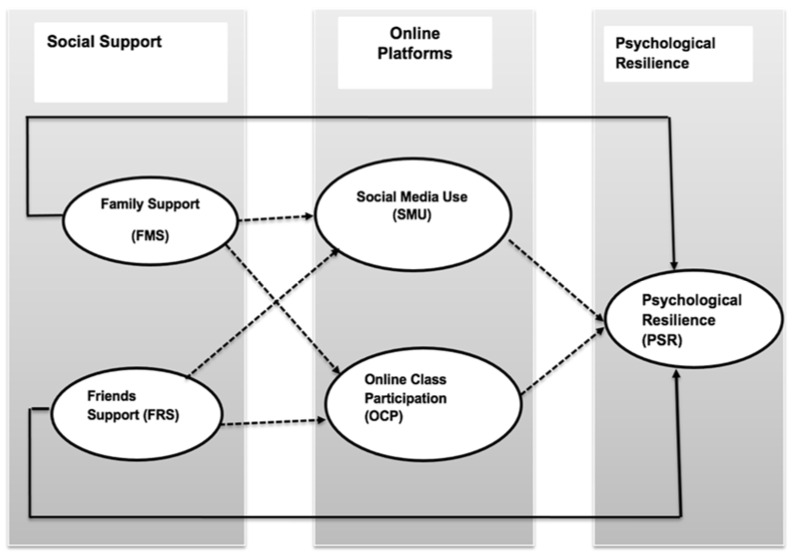
Conceptual framework.

A conceptual framework was developed based on the hypotheses of the study. Social support was divided into the exogenous constructs of family support (FMS) and friends support (FRS). Online platforms were classified into parallel mediating constructs of social media use (SMU) and online class participation (OCP). Psychological resilience was considered as an endogenous construct. The overall possible relationships of the constructs are given in the conceptual framework shown in Figure 1. 

## 3. Research Methods

### 3.1. Research Approach 

A survey analysis approach was used in this study for three reasons: First, the effect of social support through online platforms on psychological resilience among pre-service SNE teachers requires a self-reported questionnaire to understand the psychological well-being of the respondents during the COVID-19 pandemic lockdown. Second, a larger sample size was needed to generalize the results to the population of the study. Third, we used personal contacts to collect data from the target population.

### 3.2. Questionnaire Development

There were three major sections in the questionnaire. Part one consisted of the questionnaire title and description, including the aims of the study, ethical statements, and respondent consent for voluntary participation in the study. Part two contained demographic information. Part three contained a total of 19 questionnaire items on a Likert-type scale ranging from 1 = strongly disagree to 5 = strongly agree. The questionnaire items were further categorized into five factors: social media use (SMU), family support (FMS), friends support (FRS), online class participation (OCP), and psychological resilience (PSR). The questionnaire was written in English, as the respondents all understand the English language well. The reliability and validity of the questionnaire was established through a pilot study. A total of 20 PhD students and 10 university teachers responded to the pilot study. The reliability of the questionnaire was found to have a Cronbach’s alpha above the threshold of 0.8. The respondents also commented on the validity of the questionnaire content, suggesting some changes that were incorporated into the questionnaire before its mass distribution. 

### 3.3. Sample and Data Collection

An online calculator [38] was used to compute the sample size required for research that utilizes a structural equation model (SEM), given the 5 observed constructs and 19 latent indicators, the anticipated effect size was 0.2, the statistical power level was 0.8, and the probability level was 0.05. The calculator computed the required sample size as 376 based on the recommendation of the researchers [39,40]. The research ethics committee approved the research plan, and proper consent was obtained from the respondents for voluntary participation in the study. Pre-service SNE teachers from almost all SNE departments in the universities of Pakistan were available on a social media-based forum. A cluster purposive sampling technique was used to collect data from the pre-service SNE teachers available on mentioned social media forum who have the background to work in allied health sciences. The Qualitrics.com website was used to collect data from the participants, as this UK-based third-party website is well-known in Pakistan for data collection. A total of 450 questionnaires were distributed. Responses were received from 390 participants; 13 questionnaires were discarded as they were incomplete. The response rate of the completed surveys was 84%. 

### 3.4. Construct Measurement 

The questionnaire was adopted from a study [37] and comprised five constructs. Family support and friends support were considered exogenous latent constructs; online class participation and social media use were considered mediating constructs; and psychological resilience was considered an endogenous latent construct. 

#### 3.4.1. Family Support (FMS)

The three items in the family support construct were adopted and modified from a study [37]. The sample items were ‘Your parents ask you about your studies’, ‘Your parents are proud of you when you do something great’, and ‘Your parents give you some advice or support when you feel down.’ The value of Cronbach’s alpha for the construct was determined according to researchers [41,42] who indicate that a Cronbach’s alpha value (α = 0.764) for a construct above a threshold of 0.7 shows the consistency and reliability of the construct. 

#### 3.4.2. Friends Support (FRS)

The three items in the friends support construct were adopted and modified from a study [37]. The sample items were ‘You have close friends to talk to when you feel down’, ‘Your friends usually help you when you have to deal with difficult situations’, and ‘You prefer to spend your time working with friends.’ The Cronbach’s alpha value (α = 0.779) was satisfactory to confirm the reliability and consistency of the construct. 

#### 3.4.3. Social Media Use (SMU)

The social media use construct comprised four items and was adopted and modified from a study [37]. The sample items were ‘You spend time on social media every day (e.g., Facebook or WhatsApp)’, ‘Social media helps you to keep in touch with your friends and family’, ‘You use social media for academic purposes’, and ‘Social media makes you feel better when you feel bad.’ The reliability and consistency of the construct was confirmed with Cronbach’s alpha (α = 0.774). 

#### 3.4.4. Online Class Participation (OCP)

The construct of online class participation comprised three items and was adopted and modified from a study [37]. The sample items were ‘You are encouraged to participate in online classroom activities’, ‘You are given a chance to share your opinions or thoughts in online class’, and ‘You talk to your teachers about difficult situations in online classes.’ The reliability and consistency of the construct was confirmed with Cronbach’s alpha (α = 0.690). 

#### 3.4.5. Psychological Resilience (PSR)

The construct of psychological resilience comprised four items and was adopted and modified from studies [37,43]. The sample items were ‘You deal with different tasks at the same time’, ‘You believe that you can achieve your goals’, ‘You believe that you can deal with new challenges’, and ‘You believe that you can cope with stress.’ The reliability and consistency of the construct was confirmed with Cronbach’s alpha (α = 0.74), and the reliability and consistency of the questionnaire was measured with Cronbach’s alpha and found to be satisfactory.

### 3.5. Demographic 

The distribution of the sample is given in Table 1. Regarding gender, the participants were 31% male and 69% female; the participants’ ages ranged from 18 to 25 years (56%), 26 to 30 years (35%), and over 30 years (8%); the participants’ qualification distribution was BS (56%), MS/MPhil (39%), and PhD (5%); allied health work experience comprised internship (49%), less than one year (30%), one to three years (11%), three to four years (6%), and more than five years (5%), as given in Table 1.

## 4. Data Analysis and Results

SPSS (IBM, Armonk, NY, USA) and SmartPLS 3.2.8 (SmartPLS GmbH, Bönningstedt, Germany) were used for data analysis in this study. Structural equation modeling (SEM) consisted of confirmatory factor analysis (CFA), mediation analysis, and path analysis. The authors of [44] suggest a two-step procedure for the measurement of an outer model and an inner model. According to [45,46], PLS-SEM is considered the best available technique to perform multivariate analysis. 

### 4.1. Evaluation of Outer Model 

The measurement model acceptability was evaluated by measuring the consistency of the single items with relevance to their constructs, internal reliability between items, the discriminant, and convergent validity. According to researchers [47], single observed variables may retain an outer loading value of 0.40 to 0.70 and above to be included in the relevant factor. The factor loading for each item was above 0.65, fulfilling the criterion that an item should have a minimum loading of 0.4 with the relevant factor. The outer loadings ranged from 0.668 to 0.866, which was found to be satisfactory to retain the items with their relevant constructs as given in Table 2. Researchers [42,48] suggested that Cronbach’s alpha and rho alpha above a threshold of 0.7 shows that all constructs have high reliability and consistency. The Cronbach’s alpha of all constructs was observed above the given criterion of 0.7 ranging from 0.735 (Online class participation) to 0.837 (Social media use), which reflects that all the constructs were internally consistent and reliable, as shown in Table 2. The Cronbach alpha values were counter checked with rho_A values that also reflected a satisfactory reliability of the constructs ranging from 0.741 (Online class participation) to 0.84 (Social media use), as shown in Table 2. 

#### Convergent and Discriminant Validity

Item loading, composite reliability, average extracted variance, and discriminant validity was measured to assess the convergent validity. All item loadings were higher than 0.65, which reflects a high convergent validity of the constructs. Meanwhile, the composite reliability above the threshold of 0.7 and AVE values above the threshold of 0.5 [49,50,51] shows that constructs were reliable with satisfactory convergent validity, as shown in Table 2. 

This research used a heterotrait–monotrait ratio of correlations (HTMT) to measure discriminant validity. A unique approach, HTMT was introduced by Henseller et al. [52] to measure the discriminant validity of the construct. HTMT measures the similarity between two latent constructs, and the HTMT value must be less than 1 to establish discriminant validity. However, it is considered good if the HTMT value is less than 0.9 [53]. All latent constructs have shown HTMT values lower than 0.9, as given in Table 3. 

### 4.2. Evaluation of Inner Model

The constructs were proved reliable and valid. The next step was taken to measure inner model evaluation. This contains the measurement of variance inflation factor (VIF) stats, direct paths, indirect specific paths, coefficient of determination R^2^, predictive relevance, and goodness of fit index. 

#### 4.2.1. Coefficient of Determination (R^2^)

The model’s predictive accuracy was evaluated using R-square, which measures the overall variance explained and effect in a structural model. In this model, the OCP endogenous variable has shown 0.37 R^2^, PSR (R^2^ = 0.29), and Social media use (R^2^ = 0.31). Studies [44,54] have recommend that the R-square value should be greater than 0.10. All R-square values for the endogenous variables were greater than 10% and therefore satisfactory, as given in Table 4. 

#### 4.2.2. Predictive Relevance

**Figure 2:**
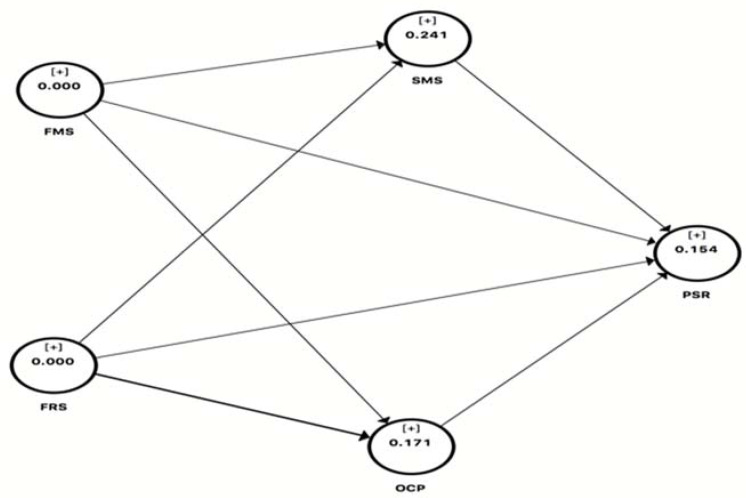
Cross-validity redundancy analysis.

Stone-Geisser’s Q^2^ value [55] is used to measure criterion predictive accuracy in addition to measuring the R^2^ values. Cross-validation redundancy analysis was used to measure the quality of the path model. Blindfolding was applied to measure the Q^2^. This represents how well a model can predict the endogenous variables. The endogenous variables must return q-square values greater than zero in SEM. Redundancy analysis has shown that Q^2^ (=1-SSE/SSO) values were greater than zero, as given in Figure 2. This shows that the predictive relevance of the model was satisfactory for endogenous variables. 

#### 4.2.3. PLS predict

The PLS predict algorithm was developed by researchers [56] as a method to train and hold the sample for PLS model estimations to generate and evaluate predictions. PLS predict was applied to assess the model’s predictivity at the level of the observed variables. A model is considered to have good predictive power if the Q^2^ values of all endogenous variables are greater than zero, as well as some or most of the root mean square error and linear regression model (RMSE_LM) values being greater than the root mean square error and partial least square (RMSE_PLS) values [57]. All the Q^2^ predict values of the observed endogenous variables were greater than zero, and RMSE (PLS-LM) values show that there is a moderate level of predictivity among relations, as shown in Table 5. 

#### 4.2.4. Goodness of Fit

The goodness of fit (GoF) is applied to verify the effectiveness of the model to explain the empirical data [58]. GoF ranges between 0 and 1 where 0.10 is smaller, 0.25 is moderate, and 0.36 is considered as substantial validation of the path model. GoF reflects the parsimony and plausibility of the model, and it is measured with a formula GoF = sqrt ((*average AVE*) ∗ (*average R^2^*)). The measurement has shown that the model was substantially fit to predict the empirical data with a GoF value of 0.44, as given in Table 6. 

Standardized Root Mean Squared Residual (SRMR) is the standardized residual that is an index between the observed and hypothesis covariance matrices [59,60]. SRMR measures the estimated fit of the given model. A path model with SRMR ≤ 0.08 is called a good fit [61]. Table 7 shows Normed Fit Index (NFI) = 0.081 and SRMR = 0.063. VIF was also observed to assess collinearity issues. Researchers [42,62] state that the VIF value should be less than the threshold of 5 in order to avoid collinearity issues. There was no collinearity issue in this case, as all inner VIF values were below the threshold of 5 [63,64], as given in Table 7. 

#### 4.2.5. Structural Model Path Coefficients

The *β* coefficients in the regression path coefficient in PLS are considered the same. *β* values were used to test the hypotheses [65]. *β* denotes the per unit variation effect of the independent variable on the dependent variable. The *t*-statistics and significance level were used to verify the *β* values. The bootstrapping procedure was used to measure the significance of the hypotheses [66]. Control variables such as age, gender, and qualification did not show a significant effect on the dependent variable PSR. The bootstrapping results for 5000 subsamples with the values of path coefficient, *t*-statistics, and significance level are shown in Table 8. 

Demographic variables such as age, gender, and qualification show no significant (*p* > 0.05) effect on endogenous latent construct psychological resilience, as given in Table 8. 

Family support has shown a significant and positive effect on psychological resilience (*β* = 0.296, *t* = 4.353, *p* < 0.001); hence, hypothesis 1 (a) was supported. Friends support has not shown an effect on psychological resilience (*p* > 0.05); therefore, hypothesis 1 (b) was rejected. Family support has shown a positive and significant effect on social media use (*β* = 0.329, *t* = 5.944, *p* < 0.001); hence, hypothesis 2 (a) was not rejected. Friends support has shown a significant positive effect on social media use (0.366, *t* = 6.383, *p* < 0.001); therefore, hypothesis 2 (b) was supported. Family support has shown a positive and significant effect on online class participation (*β* = 0.395, *t* = 7.265, *p* < 0.001); hence, hypothesis 2 (c) was supported. Friends support has shown a positive and significant effect on online class participation (*β* = 0.235, *t* = 4.047, *p* < 0.001); therefore, hypothesis 2 (d) was not rejected. Online class participation has shown a significant positive effect on psychological resilience (*β* = 0.180, *t* = 2.971, *p* < 0.003); hence, hypothesis 3 (a) was supported. Social media has shown a significant positive effect on psychological resilience (*β* = 0.146, *t* = 2.22, *p* < 0.026); therefore, hypothesis 3 (b) was supported. 

#### 4.2.6. Indirect Specific Paths

The most robust path is observed for the mediation of online class participation in the relation between family support and psychological resilience (*β* = 0.071, *t* = 2.77) at *p* < 0.005; hence, hypothesis 4 (a) was robustly supported. Online class participation has shown a significant positive mediation between friends support and online class participation (*β* = 0.042, *t* = 2.293, *p* < 0.022); hence, hypothesis 4 (b) was supported. Social media use has shown a significant and positive mediation between family support and psychological resilience (*β* = 0.048, *t* = 2.088, *p* < 0.037); hence, hypothesis 4 (c) was supported. Social media use has shown a significant positive mediation between friends support and psychological resilience (*β* = 0.053, *t* = 2.047, *p* < 0.041); hence, hypothesis 4 (d) was supported. All the mediating paths given in Table 9 are significant, as shown in Table 9. 

**Figure 3:**
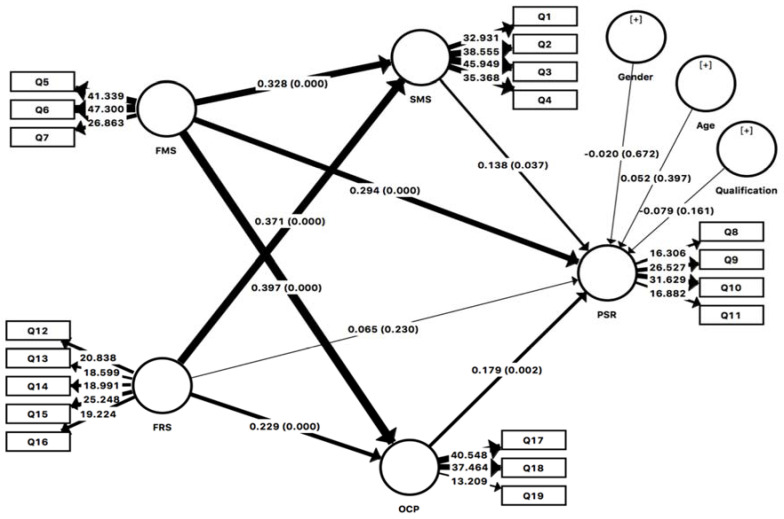
Path analysis.

Figure 3 represents the accepted paths among variables.

## 5. Discussion

RQ1. What was the impact of social support (i.e., support from family and friends) on online class participation and social media use among pre-service SNE teachers during the COVID-19 lockdown? 

The present study was conducted to explore factors that contribute toward enhancing psychological resilience among pre-service special education teachers with a background in allied health work during the COVID-19 lockdown. The SEM path constructed as a result demonstrated an interrelationship of factors influencing the psychological resilience of these pre-service teachers during the COVID-19 lockdown. This study has found that social support from friends and family boosted psychological resilience among pre-service special education teachers during the COVID-19 lockdown. 

It was found that social support from friends and family increased the students’ online class participation. Previous research has also shown that social support during the pandemic was one of the factors that prepared students to participate in online classes [67]. According to research [68], students’ parents, family, and friends encourage them to attend online classes. They not only offer emotional support to overcome anxiety, stress, and loneliness, but they also help to provide infrastructure such as mobile phones, internet access, and laptops. Online class participation helped students to connect with their classmates, friends, peers, and teachers. The availability of the students’ social circle in online environments enabled them to support each other emotionally and psychologically during the COVID-19 lockdown period, helping them stay psychologically strong. 

This research has found that social support from family and friends during the COVID-19 pandemic increased social media use, as the students’ friends, colleagues, peers, and mentors are mostly available on social media networks. These sites provide a means of connecting students with their friends [69]. Instead of face-to-face interaction during the pandemic, students used social media platforms to discuss their problems with friends. The positive effect of social support from friends and family through social media use is consistent with a previous study [29] which found a relation between social support and social media use.

RQ2. What effect did online platforms (i.e., social media and online class participation) have on psychological resilience among pre-service SNE teachers during the COVID-19 lockdown? 

Resilience is the ability to bounce back from stressful situations [70]. Findings have shown the positive effect of social media and online class participation on the participants’ psychological resilience. Larger estimated audiences and more extensive networks on social media indicated higher levels of perceived social support [30]. Findings also suggest that social network sites provide an opportunity to fulfill individuals’ psychosocial needs in a fast-paced and rapidly changing world [36,71]. 

This study has revealed that social media usage enhances psychological resilience, which is consistent with previous research [37] on whether social networks utilizing web 2.0 and online class participation could reduce feelings of loneliness and depression and boost levels of perceived social support and self-esteem in undergraduate students. 

RQ3. What was the mediating role of online platforms (i.e., social media and online class participation) in the relation between social support (i.e., from family and friends) and psychological resilience in pre-service SNE teachers during the COVID-19 lockdown? 

This study has also found that social media and online class participation played an important mediating role between support from family and friends and psychological resilience during the pandemic. Researchers [72,73] point out that social media and internet-based resources such as Twitter, YouTube, and Facebook help people to communicate and share information. Moreover, a previous study [74] found that smart phones and the availability of internet access have increased the use of social media and other online platforms, such as e-learning, among students. The fact that mobile phones continue to function even when landlines are affected by disasters means that communication/support can be maintained during a crisis. Social media plays two important roles during a crisis: It helps to access timely information from informal and official sources, and it also connects people with their loved ones and community who provide relief, help, and support [75]. The findings that social support can positively influence resilience, subjective well-being, and psychological health through online resources such as social media and internet are consistent with those of previous studies [76]. Another study [31] concluded that social support gives hope that charges a positive psychological energy; therefore, pre-service special education teachers with higher dispositional hope were more motivated and were more likely to create pathways for attaining their desired goals. This positive psychological energy enables students to deal with stressful situations, such as those created by disturbed routines due to the COVID-19 pandemic, making them more resilient and strengthening their willpower to adapt to the changing learning pattern as well as living environments [77]. Researchers [78] posited that the individual capacity to demonstrate psychological resilience also varies from person to person. A previous study [79] suggested that some people react to stress and traumatic events by withdrawing their psychological defense and succumbing to anxiety and depression; on the other hand, some people overcome negative feelings relatively quickly, resuming their normal life routines or adjusting to change [80]. It was further reported that average resilience was higher among those who perceived better social support from their families, friends, and other important people in their lives. Moreover, positive emotions and perception of life satisfaction also increased psychological resilience [21].

## 6. Conclusions

This study aimed to understand the effect of social support from family and friends on psychological resilience among pre-service special education teachers. The study also sought to reveal the mediating role of online platforms such as social media and online class participation to link social support with psychological resilience in these pre-service teachers during the COVID-19 pandemic lockdown. Keeping in view the major objectives of the study, we set four major hypotheses. Hypothesis 1 was about the effect of family and friends’ support on psychological resilience. It was observed that family support has a direct effect on psychological resilience, but friends’ support did not influence psychological resilience directly because pre-service SNE teachers’ face-to-face interaction was cut off from their friends due to the lockdown. Hypothesis 2 was about the influence of family and friends’ support to use of social media, which was approved. Hypothesis 3 was about the influence of family and friends’ support to participate in the online class, which was approved. Hypothesis 4 was about the mediating role of online platforms (such as social media and online class participation) between social support (such as family and friends) and psychological resilience among pre-service SNE teachers, which was also approved. 

This study has shown a relationship between social support from friends and family through online class participation and social media usage and the participants’ psychological resilience, which means those students who scored higher on social media usage as well as online class participation also achieved high scores on the psychological resilience scale.

It was hypothesized that social media usage would reduce levels of depression and boost perceived social support and self-esteem among pre-service special education teachers. Findings from the parallel mediation model revealed that social media usage had a significant and positive predictive effect on the resilience and subjective well-being of pre-service SNE teachers. These findings suggest that undergraduate and postgraduate students who receive positive family support from their general use of social media and support from classmates and teachers through the formal use of specific media are more likely to be able to bounce back from stressful situations, have greater subjective well-being, and have better chances of continuing their studies.

### 6.1. Implications and Future Research

COVID-19 has entered the fourth wave in some areas of the world, proving its destructiveness. The brutal reality of the COVID-19 crisis has raised questions about the adjustments required to meet imminent teaching and learning needs to secure the future of the present generation of students. Moreover, the current scenario poses challenges not only for student teachers’ learning but also for teacher educators as well to rethink ways of (re)educating teachers to face unpredictable and unknown situations that connect with issues related to equity and social justice [81]. Higher education policymakers must ask themselves: ‘Are we ready to meet the post-COVID-19 challenges facing the education system and especially teacher education?’ [82] and: ‘If ready, how shall we meet this challenge?’ [83].

There are many reasons why e-learning is underutilized in traditional universities. The main one is that teachers are not sufficiently prepared to leverage technology for educational purposes [84]. Participation in social media networks helps pre-service teachers to develop mindsets and skills for ICT usage. It helps to improve their confidence and overcome their different professional development challenges. In special education, the use of social media and technology is justified and befitting to the educational needs of the students. For example, the autism spectrum is one of the many areas where special education teachers may utilize social media to enhance the socializing capacity of students with autism. The use of social media can benefit autistic students with different ranges of autism by improving their interpersonal and social skills. Different websites, such as Habbo.com and Club Penguin, provide cyber-safety and customized programs adapted to students’ developmental level and age which can be used in special education classes. Many ordinary tasks that occur online, such as searching for a job and uploading a résumé, make the use of social media a necessity for special education students so that they do not lack the skills to become full members of society or miss opportunities to progress academically.

The implications and effects of the pandemic on education, and particularly special education, are still unknown, yet it seems certain that they will be quite challenging for educators and learners in more fragile and unstable contexts [81,82]. Hence, it is imperative for higher education policymakers to devise policies to formally incorporate social media into teaching–learning processes [83]. 

Pre-service special education teachers can receive social support from friends and family members, and this encourages them to participate in online classes. It also fosters greater social media usage. Online class participation and social media usage not only help students to enhance their psychological resilience, they also mediate the social support available from family and friends to enhance their psychological well-being. 

It is a proven fact that ICT resources and online learning play an essential role in improving students’ learning outcomes [85], but they are still under-utilized in traditional university settings [86]. According to a study [87], ICT devices and assistive technology (AT) services have been mandatory for the learning of students with disabilities for several years, and teachers are perceived as digital learners [88]. However, due to the unavailability of specialized programs in online learning technologies in teacher education, it has become difficult to find teachers trained to deliver online learning in an emergency, which affects student learning.

It is recommended that research be conducted to develop curricula and teacher training programs to increase social media usage for educational purposes among pre-service special education teachers. Teachers must be trained to adapt curricula to include social media use for special needs children to be included in mainstream education. It has been seen that people can be at greater risk of developing various mental health problems following traumatic stress. Therefore, proactive measures must be taken to sustain the psychological health of higher education students, faculty, and staff. Universities and other higher education institutions should introduce an online psychological support service for student psychological counseling during an emergency such as the current pandemic. 

It is suggested to conduct future research on the topics that how to adapt teacher education instructions and curriculum utilizing a blend of social media and e-learning platforms to enhance the sociability of the pre-service teachers during crises. It would help teacher education institutions to devise instructional policies to continue education by taking care of the psychological well-being of pre-service SNE teachers. A breed of SNE teachers who are psychologically resilient and well versed with innovative pedagogical skills would guarantee continuing the teaching–learning process in their professional field during any future crises. 

### 6.2. Limitations of Research

It was not possible to visit teacher education departments to collect data from the target audience due to the COVID-19 lockdown. Therefore, this study collected the data from the pre-service teachers who were available on a specific social media platform. Since the respondents of the survey belong to different geographical regions of Pakistan, the results of the study can be generalizable to pre-service SNE teachers enrolled in teacher education departments of Pakistan. 

## Figures and Tables

**Table 1:** Distribution of sample characteristics.

Characteristics	N	%
Gender		
Male	117	31%
Female	260	69%
Age		
18 to 25	213	56%
26 to 30	133	35%
Over 30	30	8%
Qualifications		
BS	212	56%
MS/M Phil	146	39%
PhD	19	5%
Allied health work experience		
Internship	183	49%
Less than one year	113	30%
One to three years	42	11%
Three years to five years	22	6%
More than five years	17	5%

**Table 2:** Reliability and convergent validity.

Construct	Item	Loading	α	rho_A	CR	AVE
Social media use	Q1	0.839	0.837	0.84	0.891	0.671
	Q2	0.836				
	Q3	0.830				
Family support	Q4	0.824	0.764	0.765	0.864	0.68
	Q5	0.866				
	Q6	0.783				
Psychological resilience	Q7	0.800	0.74	0.747	0.836	0.562
	Q8	0.706				
	Q9	0.693				
	Q10	0.793				
Friends support	Q11	0.721	0.765	0.769	0.85	0.587
	Q12	0.711				
	Q13	0.726				
	Q14	0.749				
	Q15	0.695				
Online class participation	Q16	0.838	0.735	0.741	0.85	0.654
	Q17	0.824				
	Q18	0.668				

**Table 3:** Heterotrait–monotrait ratio of correlations (HTMT).

Constructs	FMS	FRS	OCP	PSR
Family support				
Friends support	0.667			
Online class participation	0.662	0.561		
Psychological resilience	0.631	0.454	0.549	
Social media use	0.639	0.659	0.493	0.466

**Table 4:** Coefficient of determination (R^2^).

Endogenous Constructs	R-Square Adjusted
Online class participation	0.37
Psychological resilience	0.29
Social media use	0.31

**Table 5:** PLS predict.

Items	RMSE PLS	Q² Predict	RMSE LM	RMSE (PLS-LM)
Q1	1.160	0.173	1.122	0.038
Q2	1.102	0.255	1.113	−0.011
Q3	1.044	0.286	1.063	−0.019
Q8	0.987	0.089	0.99	−0.003
Q9	0.902	0.158	0.896	0.006
Q10	0.867	0.174	0.857	0.010
Q11	1.046	0.076	1.04	0.006
Q17	0.997	0.238	0.953	0.044
Q18	0.903	0.222	0.917	−0.014
Q19	1.152	0.036	1.12	0.032

**Table 6:** The goodness of fit index.

	Average Variance Extracted (AVE)	R-Square
	0.68	
	0.587	
	0.654	0.31
	0.562	0.29
	0.671	0.37
Average values	0.63	0.32
GoF	0.44	

**Table 7:** VIF stats and model fitness.

VIF Stats	OCP	PSR	SMS	Model Fit
Family support	1.352	2.102	1.352	SRMR = 0.063 NFI = 0.81
Friends support	1.352	1.620	1.352	
Online class participation		1.520		
Social media use		1.663		

**Table 8:** Direct paths.

Hypotheses	*β*	*t*-Stats	*p*-Values	Status
Age → psychological resilience	0.054	0.857	0.391	Rejected
Gender → psychological resilience	−0.025	0.473	0.636	Rejected
Qualification → psychological resilience	−0.078	1.423	0.155	Rejected
H1 (a) family support → psychological resilience	0.296	4.353	0.000	Not rejected
H1 (b) friends support → psychological resilience	0.064	1.133	0.257	Rejected
H2 (a) family support → social media use	0.329	6.057	0.000	Not rejected
H2 (b) friends support → social media use	0.366	6.383	0.000	Not rejected
H2 (c) family support → online class participation	0.395	7.265	0.000	Not rejected
H2 (d) friends support → online class participation	0.235	4.047	0.000	Not rejected
H3 (a) online class participation → psychological resilience	0.180	2.971	0.003	Not rejected
H3 (b) social media use → psychological resilience	0.146	2.228	0.026	Not rejected

**Table 9:** Indirect paths specific relations.

Hypotheses	*β*	*t-*Stats	*p*-Values
4 (a) Family support → online class participation → psychological resilience	0.071	2.771	0.006
4 (b) Friends support → online class participation → psychological resilience	0.042	2.293	0.022
4 (c) Family support → social media use → psychological resilience	0.048	2.088	0.037
4 (d) Friends support → social media use → psychological resilience	0.053	2.047	0.041

## Data Availability

The datasets used and/or analyzed during the current study are available from the corresponding author on reasonable request.
